# Synergistic growth, metabolic products, and matrix quantification in dual-species biofilms of oral pathogens

**DOI:** 10.1590/1678-7765-2025-0887

**Published:** 2026-06-15

**Authors:** Evelyn Giuliana Velásquez-Espedilla, Victor Feliz Pedrinha, Maricel Rosario Cardenas Cuellar, Pedro Luis Busto Rosim, Daniela Alejandra Cusicanqui Méndez, Thiago Cruvinel, Flaviana Bombarda de Andrade

**Affiliations:** 1 Universidade de São Paulo Faculdade de Odontologia de Bauru Departamento de Dentística, Endodontia e Materiais Odontológicos Bauru SP Brasil Universidade de São Paulo, Faculdade de Odontologia de Bauru, Departamento de Dentística, Endodontia e Materiais Odontológicos, Bauru, SP, Brasil.; 2 Universidade Estadual Paulista Faculdade de Odontologia de Araraquara Departamento de Dentística Restauradora Araraquara SP Brasil Universidade Estadual Paulista (UNESP), Faculdade de Odontologia de Araraquara, Departamento de Dentística Restauradora, Araraquara, SP, Brasil.; 3 Universidade de São Paulo Faculdade de Odontologia de Bauru Departamento de Odontopediatria, Ortodontia e Saúde Coletiva Bauru SP Brasil Universidade de São Paulo, Faculdade de Odontologia de Bauru, Departamento de Odontopediatria, Ortodontia e Saúde Coletiva, Bauru, SP, Brasil.

**Keywords:** Biofilms, Dental caries, Endodontics, Enterococcus faecalis, Extracellular polysaccharides, Matrix, Streptococcus mutans

## Abstract

Biofilms exhibit virulence traits that promote microbial protection, such as an extracellular matrix composed of exopolysaccharides (EPS), and contribute to host tissue damage via acid production.

**Objective:**

This study evaluated bacterial viability, biovolume, matrix (β-polysaccharides), and EPS (α-polysaccharides) formation in monospecies and mixed biofilms of *Streptococcus mutans* and *Enterococcus faecalis*, as well as pH and lactic acid production.

**Methodology:**

Biofilms were grown on dentin blocks for 7 days, and aliquots were collected for colony-forming unit (CFU/mL) counts. Subsequently, biofilms were stained with LIVE/DEAD^®^, Calcofluor White^®^, and Alexa Fluor 647–dextran conjugate^®^ to assess bacterial viability, matrix formation, and EPS formation, respectively, using confocal laser scanning microscopy (CLSM). Lactic acid production was determined by enzymatic spectrophotometry, and the pH of the culture medium was measured using a pH meter. Kruskal–Wallis and Dunn’s tests were used for pH, viability, biovolume, matrix, and EPS analyses, while one-way ANOVA followed by Tukey’s test was applied for CFU/mL counts and lactic acid production (*α* = 0.05).

**Results:**

Dual-species biofilms showed higher viability and biovolume than *S. mutans* monospecies biofilms (*p* < 0.05), whereas the other parameters did not differ among groups (*p* > 0.05).

**Conclusion:**

Dual-species biofilms of *S. mutans* and *E. faecalis* may represent a suitable model for investigating antimicrobial strategies, as their association increased viability and biovolume, which are relevant features in cariology and endodontics.

## Graphical abstract

**Figure:**
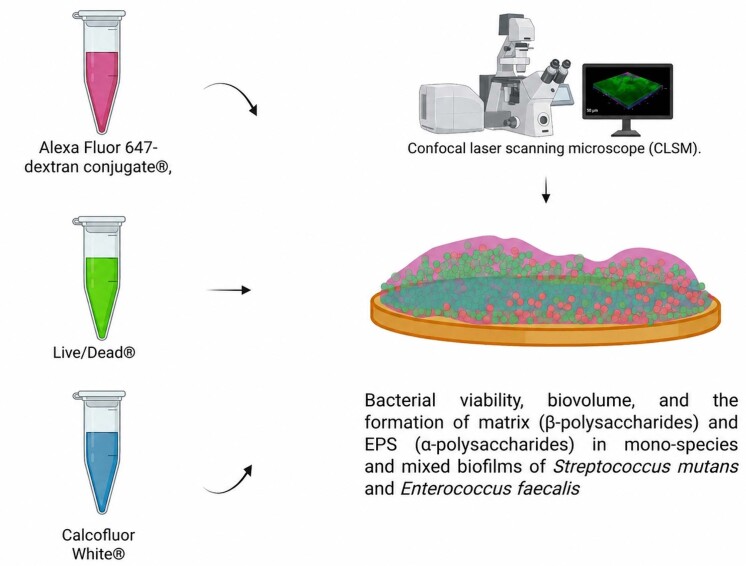

## Introduction

Biofilm formation is a key virulence factor in many infectious processes.^1^ In biofilms, microorganisms proliferate, adhere to surfaces, and co-adhere within a hydrated environment, becoming embedded in an extracellular matrix composed of polymeric substances.^2,3^ This matrix provides protection against chemical and mechanical stresses, including antimicrobial agents.^4^ It is mainly composed of exopolysaccharides (EPS), which are highly adhesive and cohesive and contribute to biofilm organization, maintenance of low pH, and modulation of gene expression, particularly in mixed-species communities.^5^

The EPS matrix comprises α-polysaccharides, β-polysaccharides, proteins, nucleic acids (eDNA), and lipids, playing a central role in maintaining biofilm structure and function. It also acts as a nutrient reservoir, supports genetic exchange and cell-to-cell communication (quorum sensing), and increases resistance to antimicrobial agents compared with planktonic cells. Consequently, biofilm control strategies may target not only bacterial viability but also matrix disruption to enhance antimicrobial penetration.^6-8^

The oral cavity harbors approximately 10^1^⁰ microorganisms that, under normal conditions, do not damage dental tissues.^9^ However, diseases such as dental caries^10^ and root canal infections^11^ are associated with microbial biofilms and their metabolic activity.

*Streptococcus mutans* (*S. mutans*) is a facultative Gram-positive bacterium strongly associated with cariogenic biofilms and also detected in infected root canals, particularly in cases of pulpal inflammation and necrosis.^5,7,12^ It exhibits several virulence traits, including adhesion, biofilm formation, acid production, and tolerance to low pH.^5,7,12,13^ Its capacity to synthesize extracellular polysaccharides plays an important role in biofilm matrix formation and structural organization, especially in mixed-species communities.^14-16^

*Enterococcus faecalis* (*E. faecalis*) is a facultative Gram-positive anaerobic bacterium commonly isolated from persistent infections associated with endodontic failure.^17^ Under biofilm conditions, *E. faecalis* exhibits enhanced virulence traits,^18^ including a high capacity to survive in unfavorable environments.^19^ In addition, it demonstrates the capacity to penetrate deeply into dentinal tubules, adhere to dentinal collagen,^20^ produce lactic acid,^21^ and form an EPS matrix, similarly to *S. mutans*.^22^ Most studies on *E. faecalis* biofilm characteristics have been conducted using monospecies models.^17,23-25^

Monospecies models allow the evaluation of specific microbial behavior but do not fully represent the natural oral environment, where microorganisms exist in complex communities rather than in isolation.^26^ However, oral infections are characterized by polymicrobial biofilms, and experimental models are required to investigate microbial behavior under controlled conditions.^25-27^

Standardized biofilm models may provide a simplified and controlled approach to evaluate ecological interactions between microorganisms, including cooperation, competition, and structural organization within the biofilm matrix.^1-5,28^ Considering that *S. mutans* is commonly associated with biofilm establishment and matrix production,^14-16^ whereas *E. faecalis* is frequently related to persistence in endodontic infections,^17^ the interaction between these species remains insufficiently understood. For this reason, a dual-species model represents a relevant experimental platform to investigate how these microorganisms interact and influence biofilm structure and viability.

Therefore, this study aimed to investigate the virulence characteristics of *S. mutans* and *E. faecalis* in monospecies and dual-species biofilms. The null hypothesis was that bacterial association would not influence biofilm parameters, including bacterial viability, biovolume, matrix content (β-polysaccharides), EPS (α-polysaccharides), pH changes, and lactic acid production.

## Methodology

Reference strains of *Enterococcus faecalis* (ATCC 29212) and *Streptococcus mutans* (ATCC 25175) were used. Colony morphology and Gram staining were periodically assessed throughout the experiment to confirm strain purity. The microorganisms were cultured in brain heart infusion (BHI) broth (Difco, Detroit, MI, USA) supplemented with 1% glucose and 1% sucrose, via successive subcultures. The culture medium was adjusted to a pH of 7.3 prior to incubation. Bacterial suspensions were standardized based on absorbance values obtained using an SF325NM spectrophotometer (Bel Photonics do Brasil Ltda., Osasco, SP, Brazil) to a final concentration of 3 × 10⁸ colony-forming units (CFU)/mL for both strains.

The BHI broth was supplemented with 1% sucrose and 1% glucose to support biofilm development and metabolic activity. This combination was selected based on pilot experiments and aimed to simulate carbohydrate availability in the oral environment. Sucrose plays a key role in extracellular matrix formation, particularly for *S. mutans*, as it serves as a substrate for glucosyltransferase enzymes involved in the synthesis of extracellular polysaccharides.^26,29^ In contrast, glucose serves as a readily metabolizable carbon source, supporting bacterial growth and metabolic activity for both *S. mutans* and *E. faecalis*.^30^

### Sample preparation

Recently extracted bovine incisor teeth, obtained by donation from a slaughterhouse, were stored in 0.1% thymol solution at 4°C. In total, 50 dentin blocks were prepared using a 5.0 mm trephine bur (Härte Surgical Instruments, Ribeirão Preto, SP, Brazil) mounted on a handpiece (KaVo Kerr, Joinville, SC, Brazil) under irrigation. To obtain standardized specimens, dentin block surfaces were sequentially polished, as previously described.^31^

To remove the smear layer, the specimens were immersed in an ultrasonic bath containing 17% ethylenediaminetetraacetic acid (EDTA), 2.5% sodium hypochlorite, and 5% sodium thiosulfate (Fórmula e Ação, São Paulo, SP, Brazil) for 5 minutes each. Subsequently, the blocks were rinsed with distilled water and sterilized at 121°C for 20 minutes. The distilled water was discarded prior to the experiments.

### Monospecies biofilm growth

All microbiological procedures were performed in a laminar flow chamber under aseptic conditions (VecoFlow Ltda., Campinas, SP, Brazil). For *S. mutans* or *E. faecalis* monospecies biofilms, 15 µL of each frozen stock culture was separately reactivated in 3 mL of sterile BHI broth supplemented with 1% glucose and 1% sucrose and incubated at 37 °C for 24 h.^32^ Successive subcultures and inoculum standardization were performed to obtain a final density of 3 × 10⁸ CFU/mL.

Sterilized dentin blocks were placed at the bottom of the wells of 24-well plates, and 100 µL of the standardized inoculum was added to each well containing 1.5 mL of BHI broth supplemented with 1% glucose and 1% sucrose. The plates were incubated at 37 °C, and the culture medium was renewed daily for 7 days to allow biofilm growth.^32^

### Dual-species biofilm growth

Sterilized dentin blocks were placed at the bottom of the wells of 24-well plates for biofilm development. After inoculum standardization as previously described, 100 µL of *S. mutans* inoculum was added to each well containing 1.5 mL of BHI broth supplemented with 1% glucose and 1% sucrose and incubated at 37 °C for 24 h.

After this initial incubation period, the culture medium was renewed, and 100 µL of standardized *E. faecalis* inoculum was added to each well. The culture medium was subsequently replaced daily, and the biofilms were maintained for a total period of 7 days.

### Confocal laser scanning microscopy

**Figure 1:**
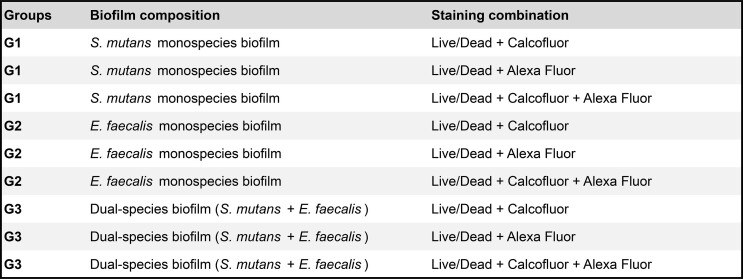
Distribution of experimental groups according to biofilm type (*Streptococcus mutans, Enterococcus faecalis*, and dual-species) and staining combination used for confocal laser scanning microscopy (CLSM), including Live/Dead®, Calcofluor, and Alexa Fluor combinations.

Specimens were randomly distributed into three groups according to biofilm growth (*n* = 15 per group): *S. mutans* monospecies biofilm, *E. faecalis* monospecies biofilm, and dual-species (*S. mutans* + *E. faecalis*) biofilm. Each specimen was considered an independent experimental unit, and biofilm formation was performed under standardized conditions across all experimental runs. Within each group (*n* = 15), specimens were further allocated into subgroups (*n* = 5) according to the staining combinations used for confocal laser scanning microscopy (CLSM) analysis: LIVE/DEAD + Calcofluor, LIVE/DEAD + Alexa Fluor, and LIVE/DEAD + Calcofluor + Alexa Fluor. The distribution of experimental groups and staining protocols is presented in Figure 1.

After the incubation period, dentin blocks containing biofilms were removed from the wells and washed twice with saline solution to remove residual culture medium and loosely adherent planktonic bacteria. The staining procedure was performed as follows: 10 µL of the LIVE/DEAD^®^ BacLight bacterial viability kit (Invitrogen Molecular Probes, Eugene, OR, USA) was applied to each specimen for 10 minutes to assess biovolume and membrane integrity. Subsequently, biofilms were stained with 10 µL of Calcofluor White M2R dye (Merck, Darmstadt, Germany) for 1 minute to identify extracellular matrix components (β-polysaccharides), followed by 10 µL of Alexa Fluor 647–dextran conjugate (Molecular Probes Inc., Eugene, OR, USA) for 1 minute to identify EPS components (α-polysaccharides). All staining procedures were performed under dark conditions.

The LIVE/DEAD^®^ kit contains SYTO 9, which stains bacterial cells with intact membranes (green fluorescence), and propidium iodide, which penetrates cells with compromised membranes (red fluorescence). Calcofluor White M2R is a fluorescent blue dye used to stain β-polysaccharides of the extracellular matrix, whereas Alexa Fluor 647–dextran conjugate is a fluorescent magenta dye used to label α-polysaccharide components of the EPS incorporated into the biofilm matrix.

Specimens were mounted on glass slides with immersion oil and observed using a Leica TCS-SPE CLSM (Leica Microsystems GmbH, Mannheim, Germany), equipped with a 40× objective lens. In total, four sequential optical sections were obtained from each specimen with a step size of 1 µm and a resolution of 1024 × 1024 pixels. The images were stacked and converted into TIFF format using Leica Application Suite Advanced Fluorescence software (LAS AF; Leica Microsystems, Mannheim, Germany). Subsequently, image analysis was performed using LAS X Life Science software (Leica Microsystems GmbH, Mannheim, Germany) to quantify cells with intact (green) and compromised (red) membranes. Bacterial viability was determined based on the analysis of each biofilm layer. The percentages of bacterial viability, extracellular matrix, and EPS were estimated following a previously described method.^20^ Biovolume values were expressed in µm^3^.

### pH measurement

For this procedure, a pH meter (PG1800, Gehaka, SP, Brazil) was used. The device was calibrated using standard buffer solutions at pH 4.0 and 7.0. The culture medium from each well of 24-well plates containing biofilms on dentin specimens was collected daily for 7 days and transferred to 2 mL microtubes. The electrode was positioned at the bottom of the sample to perform the measurements. Before each measurement, the electrode was rinsed with deionized water and gently dried with a disposable tissue.

### Lactic acid production

New dentin blocks with biofilms grown under the same conditions for each group were used (*n* = 15). At the end of the biofilm growth period, each dentin block was transferred to sterile microtubes containing 2 mL of buffered peptone water (BPW) supplemented with 0.2% sucrose and incubated anaerobically at 37 °C for 3 h.

After incubation, 200 µL aliquots from each sample were transferred to sterile microtubes, identified according to each group, and immediately placed in a water bath at 80 °C for 5 minutes to stop lactic acid production. The samples were then kept at room temperature for 30 minutes and subsequently stored at −80 °C until further analysis.

Lactic acid production was determined by enzymatic spectrophotometry.^33^ The method is based on the enzymatic conversion of L-lactate to pyruvate, coupled with the reduction of NAD to NADH, resulting in an increase in absorbance. Absorbance readings were obtained at 340 nm using a spectrophotometer (BioTek Instruments Inc., Winooski, VT, USA), based on NADH formation. The values obtained were converted into lactic acid concentrations using a standard curve ranging from 0 to 3 mM L-lactate.

### Microbiological culture evaluation

The dentin blocks, previously stored in tubes containing 2 mL of BPW supplemented with 0.2% sucrose and used for lactic acid analysis, were kept on ice and sonicated (40 mW, 1 pulse/s) for 1 minute using a Single Ultra-Sonic Cell Disruptor (Merse, Campinas, SP, Brazil). Subsequently, 100 µL of each sample was transferred to 900 µL of cysteine peptone water (CPW) medium (5 g yeast extract, 1 g peptone, 8.5 g NaCl, and 0.5 g L-cysteine HCl per liter of distilled water; pH 7.3) to perform serial dilutions up to 10^-^⁶.

Then, 50 µL aliquots of each dilution were plated onto BHI agar for the dual-species group. The plates were incubated anaerobically at 37 °C for 48 h, after which colony-forming units (CFU/mL) were determined.

For *E. faecalis* monospecies biofilms, 50 µL of each dilution was plated onto M-*Enterococcus* agar (Difco Laboratories Inc., Detroit, MI, USA). For *S. mutans* monospecies biofilms, total *mutans streptococci* were quantified by plating 50 µL of each dilution onto Mitis Salivarius-Bacitracin agar supplemented with 20% sucrose and 1% potassium tellurite (Chapman agar + 0.2 U/mL bacitracin). The plates were incubated anaerobically at 37 °C for 48–72 h. CFU counts were expressed as log₁₀ CFU/mL.

### Statistical analysis

Data distribution for each variable was assessed using the Shapiro–Wilk test. For pH and confocal microscopy analyses, the Kruskal–Wallis test followed by Dunn’s post hoc test was applied. For CFU/mL and lactic acid production, one-way ANOVA followed by Tukey’s post hoc test was used. All analyses were performed using GraphPad Prism 8.0 software (GraphPad Software Inc., San Diego, CA, USA), and the significance level was set at 5%.

## Results

**Figure 2:**
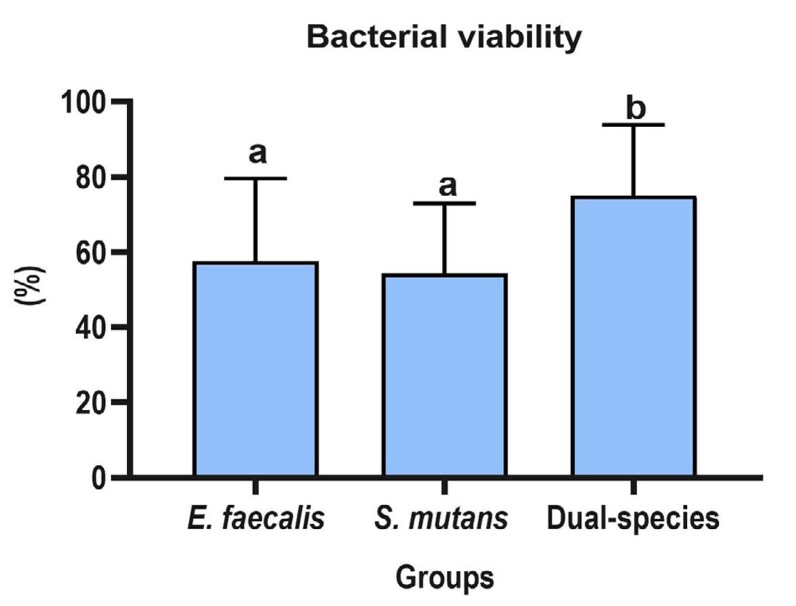
The bars indicate the median, and the vertical dashes above the bars indicate the range for the percentage of bacterial viability in different biofilms after 7 days of growth. Different superscript letters indicate a significant difference between groups by the Kruskal-Wallis and Dunn’s tests (*p < 0.05*).

Figure 2 shows bacterial viability for each biofilm group. After 7 days of growth, the percentage of viable cells in *S. mutans* and *E. faecalis* increased when grown in a mixed biofilm, with statistically significant differences compared with monospecies biofilms (*p* < 0.05).

**Figure 3:**
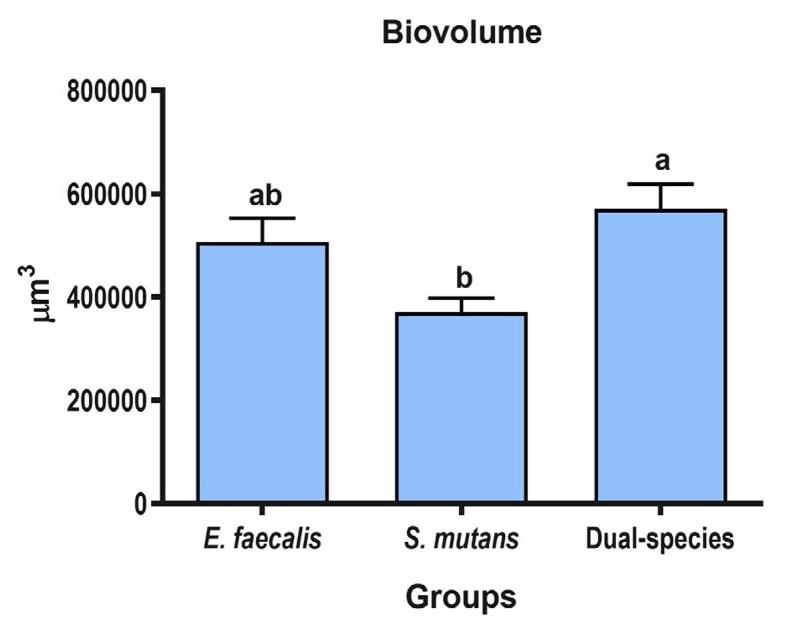
The bars indicate the median, and the vertical dashes above the bars indicate the range for biovolume values in µm3 of different biofilms after 7 days of growth. Different superscript letters indicate a significant difference between groups by the Kruskal-Wallis and Dunn’s tests (*p < 0.05*).

Figure 3 presents the comparison of biovolume among the groups. The mixed biofilm showed higher biovolume values, being significantly different from the *S. mutans* monospecies biofilm (*p* < 0.05), while the *E. faecalis* monospecies biofilm showed no significant difference (*p* > 0.05). The variability observed within groups reflects the expected biological heterogeneity of biofilm development, even under standardized experimental conditions.

**Figure 4:**
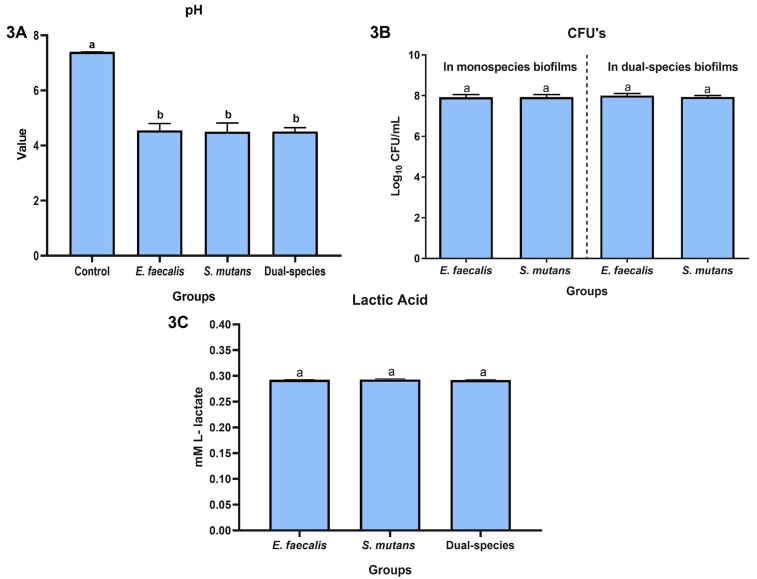
(A) The bars indicate the median, and the vertical dashes above the bars indicate the range for pH values for each biofilm and control. Comparisons were performed using Kruskal-Wallis with Dunn’s post hoc test; different superscript letters indicate significant differences between groups (*p < 0.05*). (B) The bars indicate the mean, and the vertical dashes above the bars indicate the standard deviation of CFU/mL counts for each biofilm. Comparisons were performed using one-way ANOVA with Tukey’s post hoc test; no statistically significant differences were observed. (C) The bars indicate the mean, and the vertical dashes above the bars indicate the standard deviation of lactic acid production (mM L-lactate) for each biofilm. Comparisons were performed using one-way ANOVA with Tukey’s post hoc test; no statistically significant differences were observed.

Figure 4A shows the median and range for pH values. No significant differences were observed among monospecies and mixed biofilms (*p* > 0.05), although all biofilm groups presented lower pH values than the control (*p* < 0.05). Figure 4B shows CFU/mL counts (mean ± SD), with no significant differences among groups (*p* > 0.05). Figure 4C presents lactic acid production (mean ± SD), with no statistically significant differences among the groups (*p* > 0.05).

**Figure 5:**
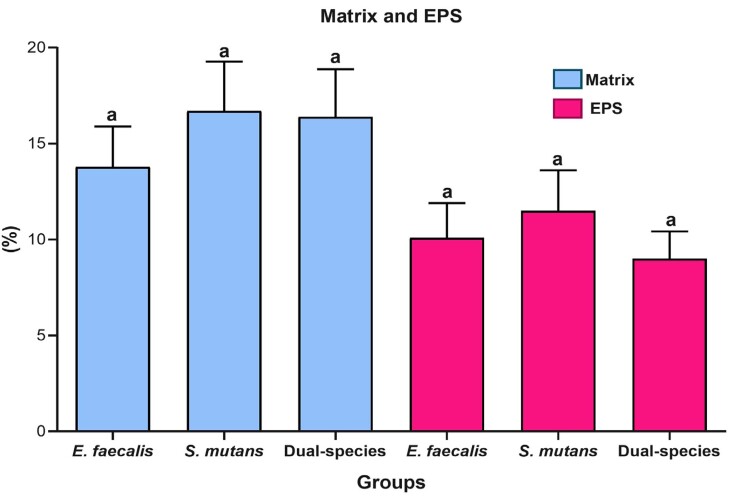
The bars indicate the median, and the vertical dashes above the bars indicate the range for percentage of biofilm matrix and exopolysaccharides (EPS) values of different biofilms after 7 days of growth. There were no statistically significant differences between groups by the Kruskal-Wallis and Dunn’s tests (*p > 0.05*).

**Figure 6:**
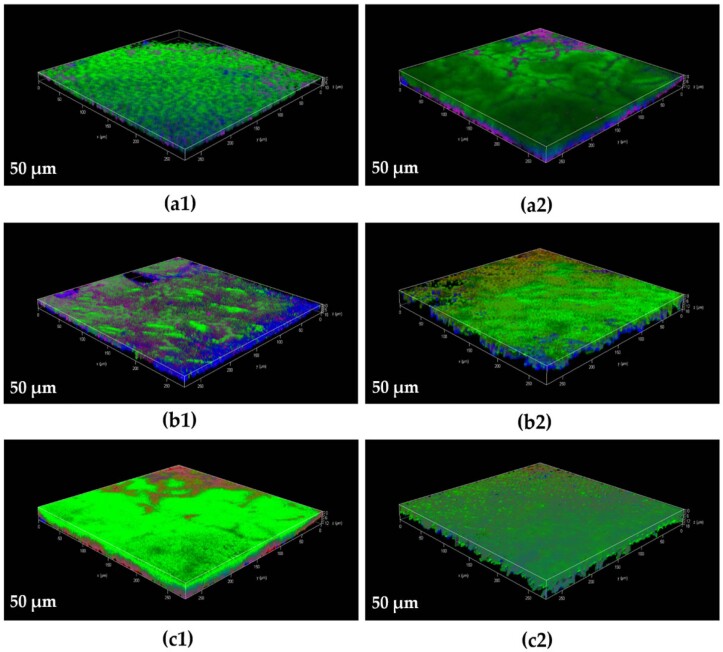
Representative confocal laser scanning microscopy images of biofilms after 7 days of growth. (a1 – a2) *Enterococcus faecalis* monospecies biofilms; (b1 – b2) *Streptococcus mutans* monospecies biofilms; (c1 – c2) Dual-species biofilms. Viable bacteria are indicated in green, and nonviable bacteria are indicated in red. Extracellular matrix components are shown in blue, and exopolysaccharides are shown in magenta. Magnification: 40×. Bars: 50.0 μm.

Figure 5 shows extracellular matrix and EPS production. No statistically significant differences were observed among the groups for matrix or EPS (*p* > 0.05), although matrix values were numerically higher than EPS. Representative images of the biofilms are shown in Figure 6.

## Discussion

The findings of this study demonstrated that the association between *E. faecalis* and *S. mutans* in dual-species biofilms formed on dentin significantly increased bacterial viability. In addition, dual-species biofilms exhibited higher biovolume compared with *S. mutans* monospecies biofilms. Based on these results, the null hypothesis was partially rejected.

Previous studies have shown that *S. mutans* biofilms may enhance *E. faecalis* biofilm formation without increasing total bacterial counts.^34^ This observation may be associated with the capacity of bacterial cells to enter a stationary or viable but non-culturable (VBNC) state, rendering them undetectable by conventional culture methods.^35^ Therefore, in addition to CFU/mL counts, the present study employed CLSM combined with LIVE/DEAD staining to assess bacterial viability based on membrane integrity.^36^

While CFU counts showed no significant differences between mono- and dual-species biofilms, CLSM analysis revealed higher viability in dual-species biofilms. This apparent discrepancy can be explained by methodological differences, as CLSM detects cells with intact membranes, including those that may not be culturable under standard conditions,^36^ whereas CFU enumeration reflects only cells capable of proliferation.^35^ Together, these findings indicate that bacterial association did not reduce the number of detectable cells but influenced biofilm viability patterns.^35,36^

Although monospecies biofilms offer experimental reproducibility, natural biofilms are multispecies communities in which microorganisms interact in synergistic, competitive, or neutral ways.^25^ In this context, dual-species models provide a controlled approach to investigate interspecies interactions. The increased biovolume and viability observed in dual-species biofilms suggest a potential synergistic effect between *S. mutans* and *E. faecalis*. However, this finding should be interpreted with caution, as it likely reflects structural and ecological interactions within the biofilm rather than direct metabolic cooperation between the species.

The selection of these microorganisms was based on their distinct ecological roles and their presence in endodontic infections. *S. mutans* is associated with biofilm establishment and extracellular matrix production,^14-16^ whereas *E. faecalis* is linked to persistence under adverse conditions.^19^ Thus, investigating their interaction may provide insights into the mechanisms involved in biofilm maturation within the root canal system while maintaining the advantages of a simplified and controlled experimental model.

To simulate aspects of biofilm development, a sequential inoculation approach was employed, allowing the initial establishment of *S. mutans*, followed by the introduction of *E. faecalis*. This strategy partially reflects ecological succession, in which early colonizers modify the physicochemical microenvironment through extracellular matrix production and metabolic byproducts, thereby facilitating the adhesion and persistence of secondary colonizers.^28^

Dentin was used as the substrate to provide a biologically relevant surface that mimics the root canal environment.^37^ Its physicochemical characteristics, including a collagen-rich matrix and dentinal tubules, influence bacterial adhesion, colonization, and persistence, making dentin-based models essential for reproducing endodontic biofilms with greater clinical relevance.^37,38^In the present study, the focus was on microbial interactions and biofilm-related parameters; therefore, structural analyses of the dentin substrate were not performed, as they fall outside the scope of the experimental objectives.

EPS (α-polysaccharides) are major components of the extracellular biofilm matrix and play a key role in biofilm formation and development.^3,39^ Regarding extracellular matrix (β-polysaccharides) and EPS (α-polysaccharides) content, the present study showed no statistically significant differences between monospecies and dual-species biofilms, although matrix values were numerically higher.

These types of analyses are challenging, as EPS formation varies according to environmental conditions, such as temperature and nutrient availability.^39^ In addition, EPS identification depends on the isolation method used. Efficient extraction from the biofilm environment remains difficult due to its complex composition, which includes a wide range of components requiring different extraction approaches. In dual-species biofilms, multiple members of the microbial community contribute distinct EPS components, which combine into a complex matrix and may persist even after the producing cells die or leave the biofilm.^39,40^

EPS in dual-species biofilms represents a complex and dynamic structure, as different microorganisms contribute distinct components that integrate into a shared matrix.^40^ Quantitative isolation of EPS is limited, as part of this fraction remains bound to bacterial cells and extraction procedures may compromise cell integrity. Therefore, staining-based approaches are considered suitable alternatives for assessing biofilm matrix components.^39^ In the present study, Calcofluor White M2R and Alexa Fluor 647–dextran were used to differentiate β-polysaccharides and EPS (α-polysaccharides), respectively. The use of these dyes enables a non-destructive evaluation of matrix composition; however, few studies have combined both markers simultaneously, highlighting the need for further investigation.

The use of sucrose and glucose in the culture medium supports both extracellular matrix formation and bacterial metabolism.^26,29^ Sucrose is directly associated with EPS synthesis by *S. mutans*, contributing to biofilm structure and adhesion,^26,29^ whereas glucose serves as a readily metabolizable energy source for both species.^30^ These conditions may also influence acid production and environmental adaptation. Notably, *E. faecalis* exhibits high tolerance to environmental stress, including acidic and alkaline conditions, which contributes to its persistence in endodontic infections.^41^

In this study, environmental pH and lactic acid production were evaluated. No significant differences in lactic acid production were observed among the groups, although both species are capable of producing acids under different metabolic conditions. Biofilms exhibited acidic pH values (approximately 4–5), despite the culture medium initially presenting a neutral pH, indicating active acid production and retention within the biofilm structure. This behavior is consistent with the capacity of *S. mutans* to metabolize sucrose and promote environmental acidification, thereby supporting biofilm establishment and stability.^12,26,29^

The presence of extracellular matrix components may contribute to the formation of localized acidic microenvironments by limiting diffusion and delaying neutralization processes.^16^ In this study, pH measurements were obtained from the spent culture medium, providing a global indicator of metabolic activity during biofilm development. However, this approach does not allow direct assessment of microscale pH gradients within the biofilm, which may vary across different layers and microenvironments.^42,43^ Therefore, the values reported here should not be interpreted as representative of local conditions at the biofilm–substrate interface, although this method is widely used as a practical indicator of acidogenic or alkalinizing metabolic processes in *in vitro* biofilm models.^29^

Lipopolysaccharide (LPS) is a well-established virulence factor in endodontic infections, particularly associated with Gram-negative bacteria and host inflammatory responses.^44^ However, the microorganisms investigated in this study, *S. mutans* and *E. faecalis*, are Gram-positive species and, therefore, do not produce LPS. Instead, their cell wall components, such as lipoteichoic acids and peptidoglycan, may contribute to host–microbial interactions.^17,21^ Accordingly, this study focused on biofilm development and interspecies interactions between these microorganisms, and endotoxin-related factors were not included, as they fall outside the scope of the experimental design.

The limitations of this study should be considered. Due to its *in vitro* design, it is not possible to fully reproduce the complexity of dental caries and root canal infections, which involve diverse microbial communities and variable environmental conditions. In addition, biofilm structure and composition may vary among clinical cases, with no single pattern representing all endodontic infections.

Despite these limitations, biofilm communities confer important adaptive advantages to microorganisms, including increased resistance to antimicrobial agents, protection against environmental stress, and enhanced pathogenic potential. In this context, the dual-species biofilm model proposed here represents a controlled and reproducible platform that may be useful for future investigations, particularly those focused on evaluating disinfection strategies in endodontics.

## Conclusions

Dual-species biofilms of *S. mutans* and *E. faecalis* exhibited increased bacterial viability and biovolume compared with monospecies biofilms. In contrast, extracellular matrix and EPS production, CFU/mL counts, pH values, and lactic acid levels did not differ among the groups. These findings suggest that the interaction between these species may promote a synergistic effect at the structural level of the biofilm; however, this effect should be interpreted with caution, as it does not necessarily reflect direct metabolic cooperation. The proposed model may contribute to a better understanding of microbial interactions in oral biofilms and serve as a reproducible platform for future studies.
